# Associations between childhood maltreatment and overt aggression in depressed adolescents: the mediating effect of alexithymia and insomnia

**DOI:** 10.3389/fpsyt.2025.1619353

**Published:** 2025-06-24

**Authors:** Jiawei Wang, Lewei Liu, Sanhua Zhou, Lili Zhao, Qianqian Wu, Tiantian Sun, Nali Zhang, Xuemei Wei, Lei Xia, Feng Zhao

**Affiliations:** 1 Department of Psychiatry, Bozhou People’s Hospital, Bozhou, Anhui, China; 2 Department of Psychiatry, The Fourth Affiliated Hospital of Anhui Medical University, Hefei, Anhui, China

**Keywords:** overt aggression, childhood maltreatment, insomnia, alexithymia, adolescents, major depressive disorder

## Abstract

**Background:**

Overt aggression may be associated with childhood maltreatment (CM), insomnia, and alexithymia, but the underlying mechanisms of these associations have not been fully explored in adolescents with major depressive disorder (MDD). Therefore, the present study aimed to deeply analyze the relationships between overt aggression and CM, insomnia, and alexithymia in adolescents with MDD, to reveal the mediating mechanisms, and to provide a theoretical basis for clinical interventions.

**Methods:**

From January 2024 to March 2025, this study included 251 adolescents with MDD. The Modified Overt Aggression Scale (MOAS), the 17-item Hamilton Depression Rating Scale (HAMD-17), the Childhood Trauma Questionnaire (CTQ), the Insomnia Severity Index Scale (ISI), and the Toronto Alexithymia Scale (TAS-20) were used to assess the adolescents’ overt aggression, depression, CM, insomnia, and alexithymia. Additionally, we employed the PROCESS macro program to examine the mediating role of insomnia and alexithymia between CM and overt aggression.

**Results:**

The prevalence of overt aggression in adolescents with MDD was 66.1%. The regression analyses showed that age (Beta = -0.761, t = -2.967, *P* = 0.003), depression (Beta = 0.183, t = 2.676, *P* = 0.008), CM (Beta = 0.132, t = 4.048, *P* < 0.001), and alexithymia (Beta = 0.092, t = 1.990, *P* = 0.048) were independent correlates of overt aggression. When CTQ subscale scores involved in the regression model, age (Beta = -0.829, t = -3.257, *P* = 0.001), depression (Beta = 0.184, t = 2.618, *P* = 0.009), emotional abuse (Beta = 0.372, t = 4.081, *P* < 0.001), and insomnia (Beta = 0.170, t = 2.054, *P* = 0.041) were independent correlates. Moreover, alexithymia and insomnia played a chain mediating role between CM and overt aggression.

**Conclusion:**

The risk of overt aggression is significantly higher in depressed adolescents and is significantly associated with CM, insomnia, and alexithymia. Additionally, insomnia and alexithymia may play a mediating role between CM and overt aggression. Given these findings, comprehensive interventions for depressed adolescents with adverse childhood experiences, insomnia, and alexithymia, should be emphasized in clinical practice to effectively reduce their risk of overt aggression.

## 1 Introduction

Major depressive disorder (MDD) is one of the most prevalent affective disorders in the adolescent population (1). In recent years, with rapid societal changes and transformations, the prevalence of MDD in adolescents has significantly increased (2). According to data from a national epidemiological survey in China, the prevalence of MDD in children and adolescents has been reported to be 2.0% (3). Not only does this disorder significantly impact adolescents’ schooling and daily lives, but it also markedly increases the risk of self-injury and suicide (4). Moreover, MDD is significantly associated with increased aggressive behaviors. For example, studies have shown that among adolescents with MDD, there is a high prevalence of aggressive behaviors, which are closely related to the severity of depressive symptoms (5). More importantly, individuals’ aggressive behaviors have been shown to significantly predict suicide attempts and deaths by suicide (6, 7). However, research on aggressive behaviors in adolescents with MDD remains limited. Therefore, it is particularly important and urgent to thoroughly investigate the current status and underlying mechanisms of aggression in this population.

Childhood maltreatment (CM) is typically defined as a serious adverse experience that an individual encounters during childhood, primarily including abuse and neglect (8). The prevalence of CM varies significantly across different populations. A large-scale survey conducted in 17 secondary schools in China found that the prevalence of CM among the general adolescent population was 34.8% (9). In contrast, the detection rate of CM in adolescents with MDD exceeds 80% (10). And, patients with MDD who have experienced traumatic childhood events tend to present a more complex set of clinical features, including an earlier age of onset of depressive symptoms, a higher frequency of relapses, a more severe and prolonged course of symptoms, and a generally poorer prognosis (11–13). Additionally, there may be connections between CM and individuals’ aggressive behaviors. For example, a cross-sectional study of Chinese children showed that psychological abuse in childhood was significantly associated with increased aggressive behaviors (14). At the physiological level, CM can disrupt specific brain structures and functions, increasing an individual’s susceptibility to aggression (15). This, in turn, can lead to adverse health outcomes and behavioral problems in adolescents, such as impulsive behaviors and violent tendencies. Nevertheless, the underlying mechanisms of the association between CM and aggression in adolescents with MDD remain incompletely understood. Given the high prevalence of CM and aggression in this population, examining the psychological and behavioral mechanisms underlying this link is crucial. Such research will help provide more specific targets for clinical practitioners to better identify and intervene in aggressive behaviors, ultimately improving outcomes for these adolescents.

Alexithymia and insomnia may play a significant role in the association between CM and aggression. Alexithymia, as an emotional processing deficit, is characterized by an individual’s difficulty in accurately identifying, differentiating, and expressing their emotional states (16). Notably, the prevalence of alexithymia is significantly higher in patients with psychiatric disorders, particularly those with depression (17). The development of alexithymia is a complex process whose roots can be traced back to an individual’s childhood. According to the Emotion Regulation Theory, traumatic childhood events may severely impair an individual’s ability to recognize and express emotions, and interfere with the normal development of the brain’s emotional center, thereby contributing to the development of alexithymia (18, 19). Emotion Regulation Theory further suggests that individuals with alexithymia are often unable to effectively use adaptive emotion regulation strategies (e.g., cognitive reappraisal) to alleviate internalized anger and tension when facing with stress or conflict (20). This emotion dysregulation may increase the likelihood of individuals adopting aggressive behaviors to cope with problems, thereby increasing adolescents’ susceptibility to aggression (21).

Additionally, insomnia may also play a mediating role in the association between CM and aggression. Previous studies have demonstrated that comorbid CM significantly increases the risk of insomnia in adolescents (22, 23). Insomnia not only impairs an individual’s ability to regulate their emotions, but may also contribute to increased emotional instability and irritability (24, 25). For example, a meta-analysis integrating 74 studies showed that poorer sleep quality was significantly associated with higher aggression in both children and adolescents, as well as in adults (26). Of interest, previous research has also found that alexithymia may contribute to the occurrence of insomnia in individuals (27). This suggests that alexithymia → insomnia may constitute a chain-mediated pathway in the association between CM and aggressive behaviors. However, this mechanism has not been fully explored in the population of adolescents with MDD.

Therefore, the aim of the present study was to analyze factors associated with overt aggression (including socio-demographic characteristics, depressive symptoms, CM, insomnia, and alexithymia) in adolescents with MDD, focusing on whether alexithymia and insomnia mediate the relationship between CM and overt aggression. Based on the above aims, we hypothesized that: (a) there are strong associations between overt aggression and socio-demographic characteristics, depressive symptoms, CM, insomnia, and alexithymia in adolescents with MDD, and (b) alexithymia and insomnia play a chain-mediated effect in the relationship between CM and overt aggression.

## 2 Methods

### 2.1 Study design and participants

This cross-sectional study was conducted from January 2024 to March 2025 at Bozhou People’s Hospital, Bozhou, Anhui Province, China, targeting adolescent psychiatric patients.. Inclusion criteria were: (1) age 12–18 years; (2) diagnosis of MDD according to the Diagnostic and Statistical Manual of Mental Disorders, fifth edition (DSM-5); and (3) willingness to participate fully in the study. Exclusion criteria included: (1) presence of other psychiatric disorders (e.g., schizophrenia, bipolar disorder); (2) significant medical conditions (e.g., major infections, autoimmune diseases); and (3) inability to complete assessments. Ultimately, a total of 251 adolescents with MDD were included, of whom 185 (73.7%) were female. The mean age of the participants was (15.42 ± 1.55) years, the mean age at onset was (13.84 ± 1.78) years, and the mean duration of illness was (18.35 ± 16.63) months (**Table 1**).

**Table 1:** Socio-demographic and clinical characteristics of adolescents with MDD.

Variables	MDD (n = 251)
Females, n (%)	185 (73.71)
Age (years), mean (SD)	15.42 (1.55)
BMI (kg/m^2^), mean (SD)	20.96 (3.84)
Age at onset (years), mean (SD)	13.84 (1.78)
Duration of illness (months), median (P_25_, P_75_)	12.00 (6.00, 24.00)
Antidepressants, n (%)	
None	116 (46.21)
SSRIs	120 (47.81)
Others	15 (5.98)
MOAS total score, mean (SD)	8.29 (6.88)
Overt aggression, n (%)	
No	85 (32.57)
Yes	166 (66.13)
HAMD-17 total score, mean (SD)	21.34 (6.28)
CTQ total score, mean (SD)	52.67 (12.88)
Sexual abuse, mean (SD)	5.94 (2.37)
Emotional abuse, mean (SD)	11.72 (4.49)
Physical abuse, mean (SD)	7.36 (2.90)
Emotional neglect, mean (SD)	16.51 (4.89)
Physical neglect, mean (SD)	11.13 (3.54)
TAS-20 total score, mean (SD)	69.03 (9.02)
ISI total score, mean (SD)	13.43 (5.17)

MDD, major depressive disorder; BMI, body mass index; SSRIs, selective serotonin reuptake inhibitors; MOAS, modified overt aggression scale; HAMD-17, 17-item hamilton depression scale; CTQ, childhood trauma questionnaire; TAS-20, toronto alexithymia scale; ISI, insomnia severity index scale.

SD, standard deviation.

The study was approved by the Ethics Committee of Bozhou People’s Hospital (No. 126, 2024), and written informed consent was obtained from all participants and their parents. Furthermore, all procedures conformed to the ethical standards of the 2013 version of the Helsinki Declaration (https://www.wma.net/policies-post/wma-declaration-of-helsinki/).

### 2.2 Measuring instruments

#### 2.2.1 Socio-demographic characteristics

Socio-demographic data were obtained via a self-administered questionnaire, covering variables such as gender, age, body mass index (BMI), age at onset, duration of illness, and use of antidepressant medication.

#### 2.2.2 Overt aggression

The Modified Overt Aggression Scale (MOAS) was utilized to assess overt aggression (28). The MOAS consists of four subscales: verbal attacks, assaults against objects, assaults against self, and physical aggression toward others. Higher total score indicates more severe aggression. Based on previous studies, the MOAS total score ≥ 4 was defined as the presence of overt aggression (28, 29). Currently, the MOAS is extensively validated and widely applied among adolescents (30, 31). In our study, the standardized Cronbach’s alpha coefficient for this scale was 0.723, which suggests satisfactory reliability for this measure.

#### 2.2.3 Depressive symptoms

The severity of depressive symptoms was assessed using the 17-item Hamilton Depression Rating Scale (HAMD-17) (32). This scale comprises 17 items, scored from 0 to 76, with higher total score reflecting more severe depressive symptoms. The HAMD-17 has demonstrated strong reliability and validity, and has been widely adopted in Chinese adolescents with MDD (33, 34). In our study, the standardized Cronbach’s alpha coefficient for this scale was 0.714.

#### 2.2.4 Childhood maltreatment

The Childhood Trauma Questionnaire (CTQ) was employed to evaluate CM in patients (35). The CTQ includes 28 items: 25 clinical items and 3 validity items, divided into five subscales: sexual abuse, emotional abuse, physical abuse, emotional neglect, and physical neglect. Each subscale comprises 5 items scored on a 5-point scale, yielding a subscale score range of 5 - 25. The CTQ total score is the sum of all subscale scores, with higher values indicating more severe childhood trauma. The Chinese version of the CTQ demonstrates robust reliability, validity, and internal consistency (36). In this study, the standardized Cronbach’s alpha coefficient for the CTQ was 0.741.

#### 2.2.5 Insomnia symptoms

The Insomnia Severity Index (ISI) was utilized to assess the severity of insomnia symptoms over the past two weeks (37, 38). This scale consists of 7 items scored on a 5-point scale. The ISI total score ranges from 0 to 28, with higher scores indicating more severe insomnia. In this study, the standardized Cronbach’s alpha coefficient for the ISI was 0.795.

#### 2.2.6 Alexithymia

The Toronto Alexithymia Scale (TAS-20) was utilized to assess difficulties in identifying and expressing emotions (39). The scale consists of 20 items scored on a 5-point scale, and the total score ranges from 20 to 100, with a higher total score indicating greater difficulties in emotional expression. The Chinese version of TAS-20 is extensively validated and widely used among Chinese adolescents with MDD (40, 41). In this study, the standardized Cronbach’s alpha coefficient for this scale was 0.731, indicating acceptable internal consistency.

### 2.3 Statistical analysis

Statistical analyses were performed using SPSS 23.0. Categorical variables were described using frequency (%). Continuous variables were assessed for normality using the Kolmogorov-Smirnov test. Normally distributed data were described as mean ± standard deviation (SD), while non-normally distributed data were described using the median (quartiles) [M (P_25_, P_75_)]. In univariate analyses, Pearson or Spearman correlation analyses were used to examine the correlations between MOAS total score with socio-demographic and clinical characteristics. In multivariate analyses, the linear regression model using the “Stepwise” method was applied to identify which factors (variables with *P* < 0.05 in the univariate analyses) were independent correlates of overt aggression (the dependent variable). In addition, Pearson correlation analyses were used to explore correlations between CM, overt aggression, insomnia and alexithymia. Finally, the mediating effects of insomnia and alexithymia in the associations between CM and overt aggression was analyzed using model 6 of the PROCESS macro program. And we performed 5,000 bootstrapped samples to assess the significance of the mediation effect, and a 95% confidence interval (CI) that did not contain zero indicated that the effect was statistically significant. In the mediation analyses, we controlled for multiple potential covariates, including age, sex, and depressive symptoms. All statistical tests were considered statistically significant at *P* < 0.05 (two-sided).

## 3 Results

### 3.1 Correlations between overt aggression with socio-demographic and clinical characteristics in adolescents with MDD

In this cross-sectional study, the prevalence of overt aggression in adolescents with MDD was 66.1% (**Table 1**). Pearson correlation analyses showed that overt aggression was negatively correlated with age (r = -0.242, *P* < 0.001) and age at onset (r = -0.209, *P* = 0.001), and positively correlated with depression (r = 0.305, *P* < 0.001), CTQ total score (r = 0.350, *P* < 0.001), emotional abuse (r = 0.333, *P* < 0.001), physical abuse (r = 0.217, *P* = 0.001), emotional neglect (r = 0.243, *P* < 0.001), physical neglect (r = 0.258, *P* < 0.001), alexithymia (r = 0.246, *P* < 0.001), and insomnia (r = 0.232, *P* < 0.001) (**Table 2**).

**Table 2:** Correlations between MOAS total score with socio-demographic and clinical characteristics in adolescents with MDD.

Variables	MDD (n = 251)	
	*r*	*P*
Gender	-0.127 ^a^	**0.045**
Age	-0.242	**<0.001**
BMI	-0.110	0.081
Age at onset	-0.209	**0.001**
Duration of illness	0.050 ^a^	0.430
Antidepressants	-0.035 ^a^	0.577
HAMD-17 total score	0.305	**<0.001**
CTQ total score	0.350	**<0.001**
Sexual abuse	0.120	0.057
Emotional abuse	0.333	**<0.001**
Physical abuse	0.217	**0.001**
Emotional neglect	0.243	**<0.001**
Physical neglect	0.258	**<0.001**
TAS-20 total score	0.246	**<0.001**
ISI total score	0.232	**<0.001**

MDD, major depressive disorder; BMI, body mass index; SSRIs, selective serotonin reuptake inhibitors; MOAS, modified overt aggression scale; HAMD-17, 17-item hamilton depression scale; CTQ, childhood trauma questionnaire; TAS-20, toronto alexithymia scale; ISI, insomnia severity index scale.

Bolded *P* value: < 0.05.

a Spearman correlation analysis.

### 3.2 Independent factors associated with overt aggression by multivariate linear stepwise regression analyses

The results of multivariate linear stepwise regression analyses were summarized in **Table 3**. When CTQ total score involved in the regression model, age (Beta = -0.761, t = -2.967, *P* = 0.003), depression (Beta = 0.183, t = 2.676, *P* = 0.008), CM (Beta = 0.132, t = 4.048, *P* < 0.001), and alexithymia (Beta = 0.092, t = 1.990, *P* = 0.048) were independent correlates of overt aggression. When CTQ subscale scores (including emotional abuse, physical abuse, emotional neglect, and physical neglect) involved in the regression model, age (Beta = -0.829, t = -3.257, *P* = 0.001), depression (Beta = 0.184, t = 2.618, *P* = 0.009), emotional abuse (Beta = 0.372, t = 4.081, *P* < 0.001), and insomnia (Beta = 0.170, t = 2.054, *P* = 0.041) were independent correlates of overt aggression. In contrast, none of the other CTQ subscales reached statistical significance (*P* > 0.05) and were therefore progressively excluded from the final regression model.

**Table 3:** Independent factors associated with MOAS total score by multivariate linear stepwise regression analysis.

Variables	Beta	t	*P*
Model 1 ^b^			
Age	-0.761	-2.967	**0.003**
HAMD-17 total score	0.183	2.676	**0.008**
CTQ total score	0.132	4.048	**<0.001**
TAS-20 total score	0.092	1.990	**0.048**
Model 2 ^c^			
Age	-0.829	-3.257	**0.001**
HAMD-17 total score	0.184	2.618	**0.009**
Emotional abuse	0.372	4.081	**<0.001**
ISI total score	0.170	2.054	**0.041**

MOAS, modified overt aggression scale; HAMD-17, 17-item hamilton depression scale; CTQ, childhood trauma questionnaire; TAS-20, toronto alexithymia scale; ISI, insomnia severity index scale.

Bolded *P* value: < 0.05.

b , CTQ total score involved in the regression model;

c , CTQ subscale scores involved in the regression model.

### 3.3 Correlations between CM, overt aggression, insomnia and alexithymia in adolescents with MDD

Correlation analyses showed that overt aggression was positively correlated with depressive symptoms, CM (including emotional abuse, physical abuse, emotional neglect, and physical neglect), insomnia, and alexithymia in adolescents with MDD (all *P* < 0.05) (**Table 4**). Furthermore, CTQ total score, emotional abuse and physical neglect were significantly positively correlated with insomnia as well as alexithymia (all *P* < 0.05) (**Table 4**).

**Table 4:** Correlations between childhood maltreatment, overt aggression, insomnia and alexithymia in patients.

Variables	1	2	3	4	5	6	7	8	9	10
1. MOAS total score	1.00									
2. HAMD-17 total score	0.305 ^***^	1.00								
3. CTQ total score	0.350 ^***^	0.345 ^***^	1.00							
4. Sexual abuse	0.120	0.121	0.447 ^***^	1.00						
5. Emotional abuse	0.333 ^***^	0.280 ^***^	0.798 ^***^	0.300 ^***^	1.00					
6. Physical abuse	0.217 ^**^	0.222 ^***^	0.639 ^***^	0.309 ^***^	0.437 ^***^	1.00				
7. Emotional neglect	0.243 ^***^	0.253 ^***^	0.786 ^***^	0.098	0.510 ^***^	0.288 ^***^	1.00			
8. Physical neglect	0.258 ^***^	0.286 ^***^	0.717 ^***^	0.186 ^**^	0.369 ^***^	0.345 ^***^	0.530 ^***^	1.00		
9. TAS-20 total score	0.246 ^***^	0.300 ^***^	0.188 ^**^	0.046	0.254 ^***^	-0.001	0.138 ^*^	0.141 ^*^	1.00	
10. ISI total score	0.232 ^***^	0.400 ^***^	0.174 ^**^	0.094	0.130 ^*^	0.162 ^*^	-0.001	0.274 ^***^	0.204 ^**^	1.00

MOAS, modified overt aggression scale; HAMD-17, 17-item hamilton depression scale; CTQ, childhood trauma questionnaire; TAS, toronto alexithymia scale; ISI, insomnia severity index scale.

^*^, *P* < 0.05; ^**^, *P* < 0.01; ^***^, *P* < 0.001.

### 3.4 Mediating effects of alexithymia and insomnia in the associations of CM and overt aggression

**Figure 1:**
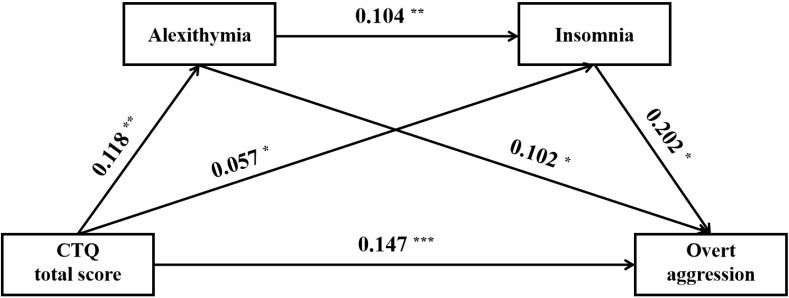
Direct and indirect relationship models of CTQ total score, alexithymia, insomnia on overt aggression. ^*^, *P* < 0.05; ^**^, *P* < 0.01; ^***^, *P* < 0.001.

**Figure 2:**
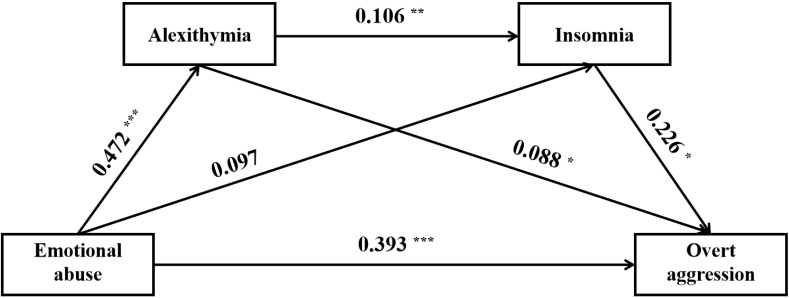
Direct and indirect relationship models of emotional abuse, insomnia, alexithymia on overt aggression. ^*^, *P* < 0.05; ^**^, *P* < 0.01; ^***^, *P* < 0.001.

**Figure 3:**
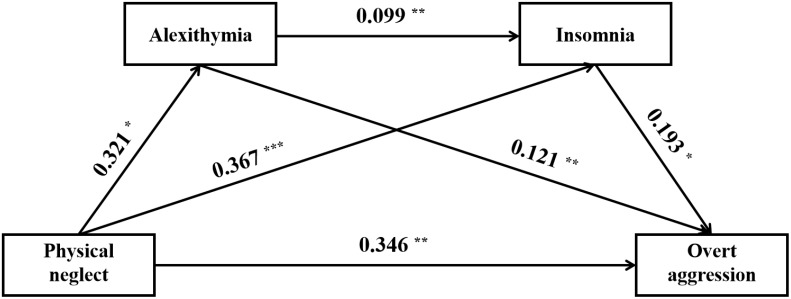
Direct and indirect relationship models of physical neglect, insomnia, alexithymia on overt aggression. ^*^, *P* < 0.05; ^**^, *P* < 0.01; ^***^, *P* < 0.001.

The results of the mediation analyses were summarized in **Table 5**, **Figures 1**–**3**. Following the PROCESS procedure, the mediating roles of insomnia and alexithymia in the associations between CM and overt aggression were analyzed, resulting in six models. In these models, overt aggression was the dependent variable, with insomnia and alexithymia as mediating variables. The independent variables were: (1) CTQ total score in Model 1, (2) sexual abuse in Model 2, (3) emotional abuse in Model 3, (4) physical abuse in Model 4, (5) emotional neglect in Model 5, and (6) physical neglect in Model 6. The results showed statistically significant indirect effects only for Models 1, 3, and 6. In Model 1, the direct effect value was 0.147 [95% CI (0.085 - 0.209)] and the indirect effect value was 0.026 [95% CI (0.008 - 0.047)]. In model 3, the direct effect value was 0.392 [95% CI (0.213 - 0.572)] and the indirect effect value was 0.075 [95% CI (0.020 - 0.143)]. In model 6, the direct effect value was 0.346 [95% CI (0.112 - 0.580)] and the indirect effect value was 0.116 [95% CI (0.035 - 0.209)]. Of note, although the indirect effect of Model 4 was not significant. However, the mediation pathway from physical abuse to insomnia to overt aggression (Model 4) appeared to be statistically significant.

**Table 5:** Mediating effects of insomnia and alexithymia in the associations of childhood maltreatment and overt aggression.

Models	Effect	Estimate	SE	95% CI	Effect size (%)
Model 1 ^d^	Total effect	0.173	0.031	0.111 - 0.235	–
	Direct effect	0.147	0.031	0.085 - 0.209	84.97
	Indirect effect	0.026	0.010	0.008 - 0.047	15.03
	CTQ total score - Alexithymia - Overt aggression	0.012	0.006	0.001 - 0.025	6.94
	CTQ total score - Insomnia - Overt aggression	0.012	0.007	0.001 - 0.027	6.94
	CTQ total score - Alexithymia - Insomnia - Overt aggression	0.003	0.002	0.001 - 0.006	1.15
Model 2 ^e^	Total effect	0.342	0.177	-0.007 - 0.692	–
	Direct effect	0.272	0.172	-0.067 - 0.610	–
	Indirect effect	0.071	0.058	-0.041 - 0.193	–
	Sexual abuse - Alexithymia - Overt aggression	0.022	0.041	-0.065 - 0.103	–
	Sexual abuse - Insomnia - Overt aggression	0.045	0.031	-0.002 - 0.119	–
	Sexual abuse - Alexithymia - Insomnia - Overt aggression	0.005	0.009	-0.015 - 0.023	–
Model 3 ^f^	Total effect	0.467	0.091	0.289 - 0.646	–
	Direct effect	0.393	0.091	0.213 - 0.572	84.15
	Indirect effect	0.075	0.031	0.020 - 0.143	16.06
	Emotional abuse - Alexithymia - Overt aggression	0.042	0.022	0.004 - 0.089	8.99
	Emotional abuse - Insomnia - Overt aggression	0.022	0.020	-0.010 - 0.067	4.71
	Emotional abuse - Alexithymia - Insomnia - Overt aggression	0.011	0.006	0.002 - 0.025	2.36
Model 4 ^g^	Total effect	0.533	0.142	0.253 - 0.813	–
	Direct effect	0.470	0.139	0.196 - 0.745	–
	Indirect effect	0.062	0.045	-0.024 - 0.156	–
	Physical abuse - Alexithymia - Overt aggression	0.002	0.029	-0.060 - 0.056	–
	Physical abuse - Insomnia - Overt aggression	0.060	0.034	0.007 - 0.136	–
	Physical abuse - Alexithymia - Insomnia - Overt aggression	0.000	0.006	-0.012 - 0.012	–
Model 5 ^h^	Total effect	0.291	0.086	0.122 - 0.461	–
	Direct effect	0.270	0.083	0.105 - 0.434	–
	Indirect effect	0.022	0.028	-0.032 - 0.079	–
	Emotional neglect - Alexithymia - Overt aggression	0.024	0.016	-0.004 - 0.059	–
	Emotional neglect - Insomnia - Overt aggression	-0.008	0.020	-0.048 - 0.031	–
	Emotional neglect - Alexithymia - Insomnia - Overt aggression	0.007	0.005	-0.001 - 0.017	–
Model 6 ^i^	Total effect	0.462	0.117	0.232 - 0.692	–
	Direct effect	0.346	0.119	0.112 - 0.580	74.89
	Indirect effect	0.116	0.044	0.035 - 0.209	25.11
	Physical neglect - Alexithymia - Overt aggression	0.039	0.023	-0.006 - 0.088	8.44
	Physical neglect - Insomnia - Overt aggression	0.071	0.035	0.008 - 0.147	15.37
	Physical neglect - Alexithymia - Insomnia - Overt aggression	0.006	0.005	-0.002 - 0.018	1.30

CTQ, childhood trauma questionnaire.

SE, standard error; CI, confidence interval; Bolded *P* value: < 0.05.

d , CTQ total score - Alexithymia/Insomnia - Overt aggression;

e , Sexual abuse - Alexithymia/Insomnia - Overt aggression;

f , Emotional abuse - Alexithymia/Insomnia - Overt aggression;

g , Physical abuse - Alexithymia/Insomnia - Overt aggression;

h , Emotional neglect - Alexithymia/Insomnia - Overt aggression;

i , Physical neglect - Alexithymia/Insomnia - Overt aggression.

## 4 Discussion

To our knowledge, this study was the first to examine the mediating roles of insomnia and alexithymia in the relationship between CM and overt aggression in adolescents with MDD. This finding enhanced our understanding of the underlying mechanisms of aggressive behaviors in this population and provided novel theoretical insights and directions for future clinical interventions and research.

In our study, the prevalence of overt aggression in adolescents with MDD was found to be 66.1%. This finding was consistent with previous research. For example, an epidemiological survey of adult patients with MDD reported that 58.7% exhibited aggressive behavior, which was strongly associated with higher depression severity and earlier age of onset (42). These observations suggested that aggression may be an important clinical feature of depression. Because of their immature psychological and cognitive development and limited self-regulation abilities, adolescents with MDD are more likely to exhibit aggressive behaviors (43). In addition, we found that the age of patients was negatively correlated with overt aggression. On the one hand, younger patients are more likely to exhibit aggressive behaviors because the brain regions responsible for emotion regulation and behavioral control, such as the prefrontal cortex, are not yet fully developed, and this underdevelopment makes it difficult for them to effectively inhibit impulsive behaviors (44). On the other hand, young patients often lack adequate psychological coping strategies and emotional expression abilities when facing depressive moods (45). As a result, they are more likely to express their distress and frustration through aggressive behaviors. In summary, overt aggression is more common in patients with MDD, particularly among adolescents. Therefore, this phenomenon warrants clinical attention, and further research into the assessment and mechanisms of aggressive behaviors in adolescents with MDD is needed.

Further analyses indicated that CM was a significant risk factor for the development of overt aggression in adolescents with MDD. Previous studies have established the strong and complex association between CM and aggression. For example, a study of Chinese adolescents found that CM has a bi-directional predictive relationship with aggression (46). Li et al.’s study based on resting-state functional magnetic resonance imaging also found that CM leads to aggressive behaviors by affecting functional connectivity between the default mode network (DMN), the cerebellar network (CON), and the dorsal attentional network (DAN) (15). Such close associations may involve complex mechanisms. On a biological level, studies have shown that CM may affect specific gene polymorphisms (e.g., the CREB1 rs4675690 T allele and the SIRT1 rs4746720 allele), which may in turn increase levels of aggression in adolescents by influencing neurobiological pathways (47, 48). A study of general adolescents also found that adverse childhood experiences may affect levels of cortisol and dehydroepiandrosterone (DHEA), which, in turn, may trigger a range of externalizing problems, such as aggressive behaviors and depression (49). Moreover, CM may also impair neuroplasticity and neurogenesis in brain circuits that regulate emotion and motivation, and this altered neurobiological basis may contribute to the increased risk of aggression in adolescents (21, 50). On a psychosocial level, Curtis et al. found that CM could influence aggressive behaviors by impairing an individual’s neurocognitive abilities (e.g., response inhibition) (51). Additionally, CM can increase the risk of aggressive behaviors in adolescents by promoting the development of specific personality traits (52, 53). A study of Chinese secondary school students found that borderline personality features were more pronounced in adolescents experiencing CM, and that these personality features were strongly associated with subsequent high levels of aggression (52). Li et al. revealed that CM significantly increased the risk of aggression in adolescents by promoting the development of callous traits (53). Additionally, another cross-sectional study conducted in China found that adolescents who had experienced childhood trauma exhibited poorer self-control, which significantly increased their susceptibility to aggressive behavior (54). Finally, of interest, the present study also found a more significant correlation between emotional abuse and overt aggression in adolescents with MDD. This finding may suggest that emotional abuse has a unique pathogenic mechanism in the development of adolescent aggression. For example, emotional abuse may more directly impair adolescents’ emotion regulation abilities and disrupt their interpersonal patterns (55, 56), thereby exacerbating the emergence of aggressive behaviors. However, this hypothesis still needs to be tested by further longitudinal studies to clarify the specific mechanisms of emotional abuse’s role in adolescent aggressive behaviors. In addition, other mediators may also play a bridging role between CM and aggression in adolescents with MDD, such as alexithymia and insomnia, which were explored in this study.

Alexithymia was shown to be a significant mediator between CM and overt aggression in adolescents with MDD in the present study. Indeed, the development of alexithymia is a long-term dynamic process whose origins can be traced back to childhood experiences, particularly those involving emotional neglect or trauma. Early adverse childhood experiences, such as emotional abuse, physical abuse, or neglect, can disrupt an individual’s normal emotional development and cognitive functioning, which can increase the risk of developing alexithymia (57, 58). For example, a prospective study in Finland found a significant correlation between adverse childhood experiences and the presence of alexithymia (59). And the association between alexithymia and aggression has also been supported by previous research. A study of young people found that alexithymia can significantly predict individuals’ aggressive behaviors (60). Individuals with alexithymia often have deficits in cognitive processing and emotion regulation, as evidenced by difficulties in recognizing and expressing their emotions, heightened reactivity to emotional stimuli, and a lack of effective emotion regulation strategies (20, 61), which may increase the risk of aggression. In addition, patients with MDD who have comorbid alexithymia are also likely to present with more severe psychopathological features, such as higher levels of anxiety, more frequent impulsive behaviors, and poorer social functioning (62, 63). These psychopathological traits not only exacerbate difficulties in emotion regulation, but also further weaken the individual’s ability to inhibit aggressive impulses, thereby contributing to the expression of aggressive behaviors in adolescents with MDD (64). Similarly, a survey of Chinese youths found that alexithymia mediated a positive association between CM and aggression (65), which further supports the findings of the present study. In conclusion, the results of the present study highlight the need to comprehensively consider the potential impact of alexithymia when intervening in adolescents’ aggressive behaviors and to develop targeted psychological interventions.

Also, the present study identified insomnia as another mediator of the link between CM and overt aggression. This finding was widely supported by existing studies. First, the association between CM and insomnia has been well established in several studies. A meta-analysis that included 26 studies noted the significant associations between CM and a range of sleep problems, including insomnia symptoms, shorter sleep duration and nightmares (66). On the one hand, adolescents experiencing CM often face many psychological stresses. For example, they may experience chronic high levels of stress and exhibit hypervigilance, anxiety, or depression, and these psychological states can significantly disrupt normal sleep rhythms, thereby increasing the risk of insomnia (67, 68). On the other hand, CM has been shown to affect the balance of neurotransmitters, such as decreasing the levels of serotonin and dopamine, which play a key role in regulating sleep-wake cycles and promoting restful sleep (69, 70). In our study, insomnia was shown to be an independent risk factor for overt aggression in adolescents with MDD, which is highly consistent with previous research. For example, a longitudinal study found that the persistence of insomnia symptoms significantly increased the likelihood of individuals developing aggressive behaviors (71). In addition, neuroimaging-based studies have further revealed the underlying neurobiological mechanisms between insomnia and aggressive behaviors. It has been found that adolescents with MDD who suffer from insomnia tend to have altered functional connectivity between the prefrontal cortex and the amygdala (72). The prefrontal cortex plays a key role in emotion regulation and behavioral inhibition, while the amygdala is strongly associated with the intensity of emotional responses (73). Abnormalities in this functional connectivity may lead to impaired emotion regulation and increased impulsive behavior, thereby increasing an individual’s susceptibility to aggressive behaviors (71).

Finally, and more critically, the present study initially revealed the chain-mediated role of narrative disorders and insomnia in the association between CM and overt aggression. This chained pathway is highly compatible with theoretical models of emotion regulation (74). Specifically, individuals who experience CM tend to have significantly weakened emotion regulation, which not only increases the risk of developing alexithymia but also exacerbates the negative accumulation of emotions, making them more susceptible to insomnia during times of stress (18, 19). Insomnia, in turn, further elevates the probability of overt aggression through the neuropsychological mechanisms described above. This finding provides new insights into the complex relationship between CM and overt aggression in patients with MDD. However, when analyzing specific sub-dimensions of CM, the results showed that only emotional abuse and physical neglect were statistically significant in mediating the effects. This finding suggested that emotional abuse and physical neglect may have a more significant impact on individuals, while the roles of other sub-dimensions may be relatively minor or not yet fully revealed. In addition, other dimensions of CM may only influence overt aggression in adolescents with MDD through a single pathway (e.g., alexithymia or insomnia). For example, in Model 4 of the present study, the mediating pathway from physical abuse to insomnia to overt aggression was found to be statistically significant, even though the overall indirect effect did not reach statistical significance. However, these findings should be interpreted with caution due to the limited number of relevant studies and the relatively small sample size of this study. Further in-depth explorations are needed in the future to more fully elucidate the mechanisms of action of these sub-dimensions and their long-term effects on individual aggression.

The limitations of our study were as follows: (1) The cross-sectional study design did not allow for causal inferences. Future studies should explore the mediating roles of insomnia and alexithymia in the relationship between CM and overt aggression using a longitudinal design. (2) The study was conducted in a single hospital in Anhui Province, China, with a small sample size, limiting the generalizability of the results. It is recommended that subsequent large-sample surveys be conducted in different regions and cultural contexts to validate the robustness of the mediation model. Additionally, future studies could consider more detailed stratified analyses of the sample based on factors such as patients’ sex, age, and disease severity to explore their potential impact. (3) Some data were based on patients’ self-reports, which may introduce recall bias, and the findings should therefore be interpreted with caution. (4) There may be other unmeasured variables that mediate the relationship between CM and overt aggression, and their omission may affect the accurate estimation of the mediating effect.

## 5 Conclusion

In this study, adolescents with MDD were found to exhibit a higher risk of overt aggression, which was significantly associated with CM, insomnia, and alexithymia. Further analyses showed that alexithymia and insomnia mediated the association between CM and overt aggression. Therefore, in clinical practice, clinicians and parents should pay attention to the adverse childhood experiences of adolescents with MDD and implement timely and targeted psychological interventions to prevent the development of alexithymia and insomnia. Through multidimensional interventions, the risk of overt aggression in adolescents with MDD may be reduced, and their psychological health and behavioral performance may be improved.

## Data Availability

The raw data supporting the conclusions of this article will be made available by the authors, without undue reservation.
