# Correction: Associations between childhood maltreatment and overt aggression in depressed adolescents: the mediating effect of alexithymia and insomnia

**DOI:** 10.3389/fpsyt.2025.1734316

**Published:** 2025-11-20

**Authors:** Jiawei Wang, Lewei Liu, Sanhua Zhou, Lili Zhao, Qianqian Wu, Tiantian Sun, Nali Zhang, Xuemei Wei, Lei Xia, Feng Zhao

**Affiliations:** 1 Department of Psychiatry, Bozhou People’s Hospital, Bozhou, Anhui, China; 2 Department of Psychiatry, The Fourth Affiliated Hospital of Anhui Medical University, Hefei, Anhui, China

**Keywords:** overt aggression, childhood maltreatment, insomnia, alexithymia, adolescents, major depressive disorder

In the **Abstract**, the research timeframe was incorrectly given as December 2024 to December 2025. This has been corrected to January 2024 to March 2025.

The research timeframe was incorrectly given as December 2024 to December 2025. A correction has been made to the section *2.1 Study design and participants*, Paragraph 1:

“This cross-sectional study was conducted from January 2024 to March 2025 at Bozhou People’s Hospital, Bozhou, Anhui Province, China, targeting adolescent psychiatric patients.”

The original version of this article has been updated.

